# Distance to innovations, kinship intensity, and psychological traits

**DOI:** 10.1371/journal.pone.0279864

**Published:** 2023-01-26

**Authors:** David le Bris, Victor Gay

**Affiliations:** 1 Toulouse Business School (TBS), University of Toulouse, Toulouse, France; 2 Toulouse School of Economics (TSE) and Institute for Advanced Study in Toulouse (IAST), University of Toulouse 1 Capitole, Toulouse, France; FAME|GRAPE, POLAND

## Abstract

Psychological traits display substantial variation worldwide. These psychological variations could be explained by the intensity of kinship ties which, we hypothesize, depends on the reception of innovations that gradually complexified family organizations. These innovations originated from several centers across the world that also spread other crucial novelties such as agriculture. Less exposed to these family innovations, areas far from centers of innovation should exhibit lower kinship intensity. Indeed, we show that distance to innovation centers is strongly associated with kinship intensity. This distance is also associated with psychological traits especially outside Western Europe in which exposure to the Church seems to play an additional role.

## Introduction

What explains the substantial variations in psychological traits observed across populations, and especially, the peculiarities that characterize Western, Educated, Industrialized, Rich, and Democratic (WEIRD) peoples [1]? Schulz et al. [2] link the origins of global psychological variations to the intensity of kinship structures as caused by the historical presence of the Western Church. Specifically, the Marriage and Family Program (MFP) diffused by the Church during the Middle Ages in Europe, which promoted the nuclear family, would have weakened pre-existing intensive kinship ties. As a result, Western populations became more individualistic, independent, and impersonally prosocial, but less conformist and in-group loyal. Key to their argument, Schulz et al. [2] assume that families exhibited intensive kinship after the Neolithic revolution until the Church undermined this model, mainly in Western Europe.

However, historical evidence suggests that the West European family was already nuclear in a context of low kinship intensity before the diffusion of the Church’s MFP. Indeed, genetic and isotopic analysis do not support the presence of high kinship intensity among Western Neolithic farmers [3–7]. In particular, the practice of cousin marriage—a key indicator of high kinship intensity according to Schulz et al. [2]—was limited, as recurring evidence of female exogamy attests. Moreover, several of these studies claim to have identified evidence of nuclear families. A rich historiography generally supports that the Roman family was nuclear [8–10] based on various sources, especially funeral epigraphy [11–13] and the analysis of Roman law [14], which inheritance rules were inconsistent with a high kinship intensity system. After the fall of the Roman Empire—though before the advent of the MFP—population censuses [15–18], analyses of house sizes [19], as well as family organizations depicted in Saints vitae [20] all suggest that the nuclear family was also dominant then, while Saint Augustine [The City of God, XV, 16] testifies that cousin marriage was already socially rejected in the fifth century (we provide further discussion of this historical evidence in the Supporting Information). In this perspective, the Church’s MFP cannot have been at the root of weak kinship in Western Europe. Rather, it might have only formalized—though potentially reinforced—practices that were already in place.

To explain the distribution of kinship structures over the world, we resort to an alternative conceptual framework: Emmanuel Todd’s diffusionist theory [21]. This theory asserts that it was successive innovations in family organizations that gradually complexified the initially nuclear family that can still be observed among hunters-gatherers: first emerged a stem family in which the eldest son remains with his parents (for instance observed in pre-industrial Japan and Germany); a second innovation enabled all married sons to remain at the parental home, forming the communitarian family (observed for instance in pre-industrial China and Russia); and a later innovation imposed members of a communitarian family to marry a relative, leading to the “Arab clan.” These successive innovations gradually intensified kinship structures throughout history.

**Fig 1:**
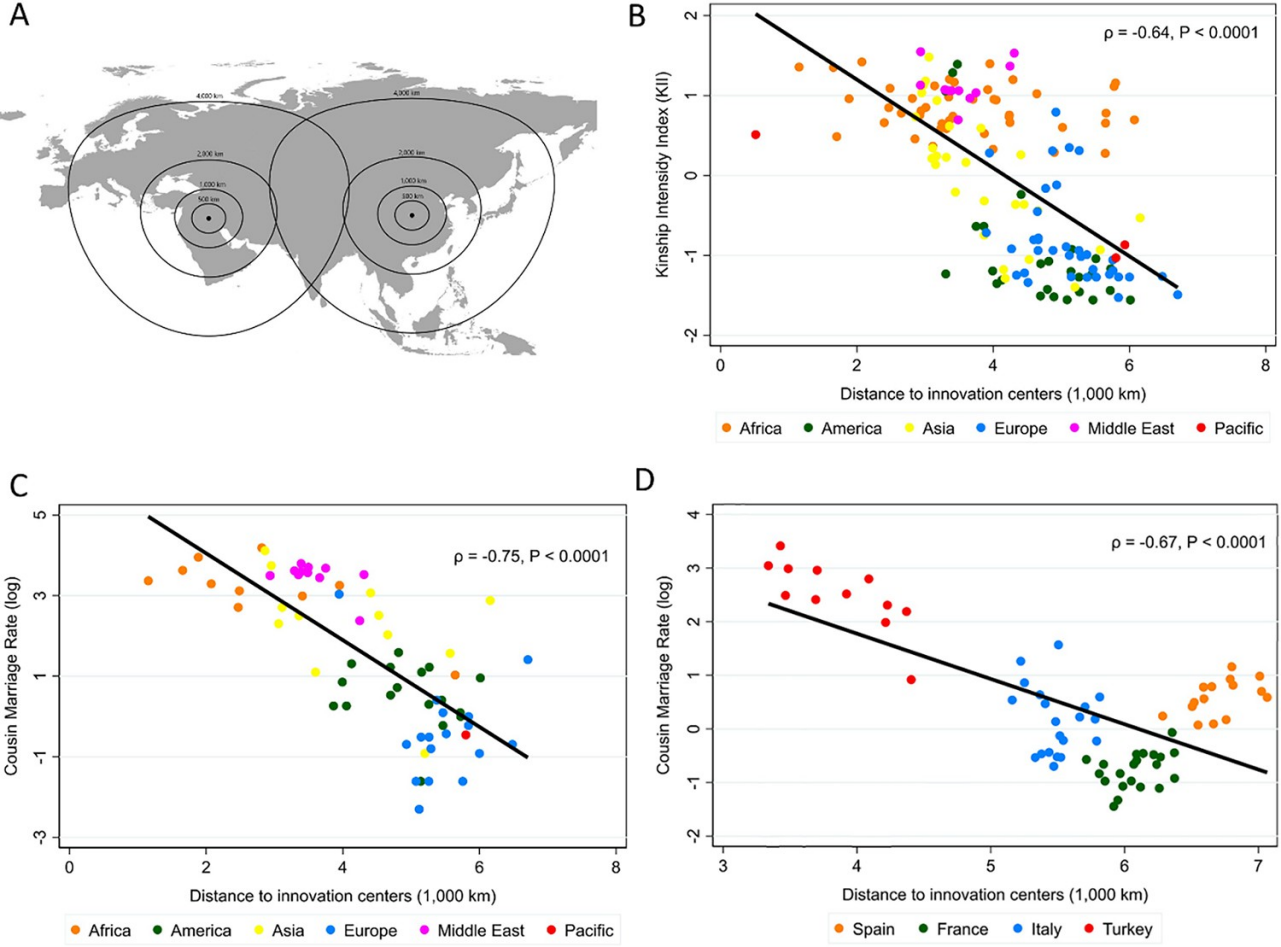
Distance to innovation and kinship intensity. Notes: Example scheme of the diffusion of kinship innovations through the case of Eurasia (**A**). Relationships between average Distance to innovation and Kinship Intensity Index and Cousin marriage rate worldwide (**B** and **C**) and Cousin marriage rate in regions of four European countries (**D**). The base map in Fig 1A is from IDRE [24] under an Open Data License.

These new family organizations emerged in several innovation centers over the world: the Fertile Crescent, Central China, the Sahel, Papua, and three locations in the Americas—these areas were also the birthplace of agriculture and in some cases urbanization, statehood, and writing. Todd’s diffusionist framework posits that family innovations then gradually spread from these locations through imitation and migration, following a pattern similar to the diffusion of writing for instance. Per the principle of conservatism of peripheral zones [21], Western Europe should have been less exposed to such innovations as it is located far from Eurasian innovation centers (Fig 1A illustrates how kinship innovations diffused along these lines in the case of Eurasia using a base map from *24*). Hence, Europe should have preserved a high prevalence of the initial family form (the nuclear family) characterized by low kinship intensity—although Western European regions closer to innovation centers, such as Southern Italy, were more exposed to family innovations than others, such as England and Scandinavia. Following this diffusionist framework, we therefore expect contemporaneous kinship intensity in a location to depend on exposure to these innovations, an exposure that we capture through the distance to the innovators in the continent.

Still, Schulz et al. [2] propose convincing empirical evidence linking kinship intensity to both historical exposure to the Church and contemporaneous psychological traits. While we acknowledge the association (claimed to be causal) between kinship intensity and psychological traits, in the framework we adopt, Church is not the fundamental cause but only one channel through which populations of Western Europe expressed their traditional family practices of low kinship intensity. We instead propose that the fundamental cause rather consists in the exposure to family innovations. Our objective in this short article is to explore whether a simple metric of Todd’s diffusionist framework—distance to innovations—exhibits some explanatory power over the diffusion of the Church’s MFP.

Relying on the rich data assembled by Schulz et al. [2], we investigate the power of this diffusionist model in explaining the joint world distribution of kinship intensity and psychological outcomes relative to Church exposure. We assess the exposure of an area (countries and regions) to innovations in family organization through its geographic distance to innovation center(s) of its continent. We then investigate the association of this distance to Schulz et al.’s [2] measures of kinship intensity and psychological traits both across countries worldwide and within European regions, comparing the respective effects of this distance and Church exposure. Finally, we offer a preliminary exploration of an alternative mechanism that could confound our insight: the direct effect on family complexity of the time since the Neolithic transition.

## Materials and methods

To test Todd’s diffusionist model, we build a measure of exposure to innovations in family organizations defined as the distance from region and countries’ administrative capital to the innovation center(s) of its continent. Though this measure is imperfect as the first appearance of an innovation and its speed of diffusion could differ across innovations and areas, it remains the most intuitive and transparent approach. To define innovations centers, we refer to the current knowledge on locations of independent appearance of agriculture [22] as they correspond to the areas where family innovations also emerged. For Oceania, we retain the distance to Kuk Swamp (Papua-New Guinea), and for North America, the distance to Eva (Tennessee). For areas that have been exposed to two centers of innovation, we retain the average of both distances as a location receives the two influences: for Eurasia, the average distance to Bagdad (Fertile Crescent) and Xi’An (Central China); for Central and South America, the average distance to Chiripa (in the Andes) and Jalisco (Mexico); and for Africa, the average distance to the Sahelian band and Cairo (agriculture was introduced there from the Fertile Crescent).

Although it remains the most intuitive and transparent approach, this measure of distance is only relevant to areas that have not been exposed to important migration flows since 1500. While it is true for most countries in Eurasia and Africa, it is not for others such as those in the Americas. To account for global historical migrations, we compute for each country an ancestry-adjusted distance (henceforth Distance) using Putterman’s [23] matrix of population movements since 1500. More precisely, we calculate the Distance to innovation of a country as the distance of its populations’ origin countries weighted by the relative share of its population of that origin. For instance, for Venezuela, we weight at 31% the average distance between Caracas to Jalisco and Chiripa as this is the share of the population of local origin, then add the Distance to innovation observed in Spain weighted at 53%, reflecting the population share of Spanish origin, and finally add in the same way the Distance of origin countries of Venezuelan populations of other origins.

## Results

### Distance to innovations as a general explanation of kinship intensity

Supporting our argument, both measures of kinship intensity proposed by Schulz et al. [2]—a kinship intensity index (KII) and the rate of cousin marriage—are strongly correlated with Distance across countries worldwide (Fig 1B and 1C). The same correlation is observed across regions of countries for which the rate of cousin marriage is available (France, Italy, Spain, and Turkey) (Fig 1D). Consistent with our interpretation of the Church as an institution formalizing pre-existing practices, Church exposure and Distance are also correlated (*ρ* = 0.53, S1 Fig in S1 Appendix), though without introducing collinearity concerns when including both variables in the analysis as Church exposure is close to zero for many areas (see VIF values in table notes).

A regression approach that includes the large set of geographical controls retained by Schulz et al. [2] reveals that Distance of each country to it(s) innovation center(s) is highly significant in explaining the distributions of the KII and of rates of cousin marriage worldwide (Table 1). Importantly, our analyses include continent fixed effects throughout to control for differences in timing of kinship innovations across continents. Our results are qualitatively similar within Eurasia—a subsample for which the location of innovation centers (the Fertile Crescent and Central China) is more firmly established—as well as within Afro-Eurasia—a subsample for which the potential inaccuracies in the ancestry-adjustment matter much less. The R-squared in these regressions further implies that Distance better explains variation in these outcomes than Church exposure does. Our results also show that Church exposure is unrelated to the distribution of rates of cousin marriage within continents, i.e., when continent fixed effects are included. Moreover, when Distance and Church exposure are jointly included, only the former comes out significant. Distance is also significantly associated with both measures of kinship intensity when focusing on countries for which Western Church exposure was almost null, inferior to 50 years (i.e., outside Western Europe), supporting Distance as a more general explanation than Church exposure. In terms of magnitudes, standardized coefficients reveal that Distance is almost always more strongly associated to the KII and cousin marriage rates than Church exposure, with standardized estimates ranging from −0.18 to −0.77 (S1 Table in S1 Appendix). Using the distance to the closest innovation center, instead of the average distance to innovation centers where several existed, generates similar though weaker results, supporting our framework in which several innovation centers affect a country (S2 Table in S1 Appendix); for instance, Afghanistan received the influence of complexifying innovations from both the Fertile Crescent and China justifying focusing on average distance rather than to the closest one. In any case, this minimum distance is more strongly associated with kinship intensity than Church exposure.

**Table 1:** Cross-country regressions of KII and cousin marriage on distance to innovations and Church exposure.

	Panel A. Kinship Intensity Index (KII)											
Sample	World			Eurasia			Afro-Eurasia			W. Church exp. < 50 years		
Distance		-0.33***	-0.30***		-0.62***	-0.66***		-0.26***	-0.24***		-0.15†	-0.13†
(in 1,000 km)		(0.06)	(0.06)		(0.09)	(0.11)		(0.06)	(0.06)		(0.07)	(0.08)
W. Church exp.	-0.12***		-0.04	-0.07†		0.02	-0.07*		-0.04	-0.86		-0.48
(in 100 years)	(0.03)		(0.03)	(0.04)		(0.03)	(0.04)		(0.03)	(0.99)		(1.14)
E. Church exp.	-0.06		-0.02	-0.01		-0.01	-0.01		-0.01	-0.07		-0.05
(in 100 years)	(0.06)		(0.06)	(0.08)		(0.08)	(0.08)		(0.07)	(0.10)		(0.09)
*N*	147	147	147	74	74	74	118	118	118	93	93	93
*R* ^2^	0.615	0.667	0.670	0.483	0.642	0.645	0.609	0.648	0.652	0.485	0.499	0.503
	**Panel B. Log Rate of Cousin Marriage**											
Sample	World			Eurasia			Afro-Eurasia			W. Church exp. < 50 years		
Distance		-0.46*	-0.41*		-0.67*	-0.64*		-0.57**	-0.53**		-0.47†	-0.49†
(in 1,000 km)		(0.18)	(0.18)		(0.33)	(0.30)		(0.19)	(0.17)		(0.25)	(0.28)
W. Church exp.	-0.15		-0.07	-0.17		-0.13	-0.19		-0.15	-0.21		1.29
(in 100 years)	(0.10)		(0.10)	(0.14)		(0.12)	(0.13)		(0.11)	(1.69)		(1.87)
E. Church exp.	0.99		0.28	0.97		-0.82	0.88		-0.92	1.67		1.53
(in 100 years)	(3.35)		(3.32)	(3.50)		(3.15)	(3.43)		(3.10)	(4.62)		(3.59)
*N*	69	69	69	41	41	41	51	51	51	33	33	33
*R* ^2^	0.742	0.766	0.768	0.742	0.773	0.783	0.754	0.786	0.795	0.260	0.338	0.346

This table reports OLS estimates from regressing country-level kinship intensity indexes (KII) in Panel A and country-level log rates of cousin marriage in Panel B on Western and Eastern Church exposure (in 100 years) and ancestry-adjusted distance to innovation (in 1,000 kilometers). All regressions include the following set of controls: ruggedness, mean distance to waterways, caloric suitability, absolute latitude, and continent fixed effects. Except for distance, data are from Schulz et al. [2]. Robust standard errors are reported in parentheses. For the World sample, VIF values when variables are jointly included are 2.10 for Distance, 3.78 for Western Church exposure, and 2.00 for Eastern Church exposure in Panel A; 3.15 for Distance, 5.69 for Western Church exposure, and 1.63 for Eastern Church exposure in Panel B.

†≤0.1

*≤0.05

**≤0.01

***≤0.001. Significance levels are based on two-tailed tests.

These results further hold when analyzing kinship intensity across 68 regions of France, Italy, Spain, and Turkey. When regressing rates of cousin marriage across regions within these countries jointly on Distance and Church exposure together with a large set of geographical controls, we find that Distance is significantly correlated with the rate of cousin marriage, while Church exposure is not (Table 2). Further including country fixed effects and controlling for exposure to the Carolingian Empire—a key factor in promoting the Church’s MFP according to Schulz et al. [2]—yields the same conclusion. Here again, the magnitudes of the coefficients on Distance revealed by standardized coefficients are much stronger than Church (S3 Table in S1 Appendix).

**Table 2:** Region-level regressions of cousin marriage on distance to innovation and Church exposure.

	Log Rate of Cousin Marriage				
Distance		-0.425***	-0.309***	-0.414***	-0.461*
(in 1,000 km)		(0.075)	(0.111)	(0.102)	(0.219)
W. Church exp.	-0.142***		-0.058	0.000	0.038
(in 100 years)	(0.032)		(0.039)	(0.036)	(0.029)
Caroligian Empire				-0.787***	-0.494***
				(0.222)	(0.167)
*N*	68	68	68	68	68
*R* ^2^	0.900	0.910	0.914	0.930	0.947
Geographic controls	Yes	Yes	Yes	Yes	Yes
Country FE	No	No	No	No	Yes

This table reports OLS estimates from regressing region-level log rates of cousin marriage on Western Church exposure (in 100 years), exposure to the Caroligian Empire, and ancestry-adjusted distance to innovation (in 1,000 kilometers). Included are regions of Spain, France, Italy, and Turkey. All regressions include the following set of controls: terrain ruggedness, distance to the coast, caloric suitability, absolute latitude, precipitation, temperature, elevation, presence of river or lake, irrigation potential, caloric suitability for oats and for rye. Except for distance, data are from Schulz et al. [2]. Robust standard errors are reported in parentheses.

†≤0.1

*≤0.05

**≤0.01

***≤0.001. Significance levels are based on two-tailed tests.

Our analyses thus suggest that Distance to innovations constitutes a more robust and general explanation of the distribution of kinship intensity than Church exposure as observed both across countries worldwide and across regions within European countries.

### Distance to innovations and Church exposure as explanations of psychological traits

Can Distance to innovations further explain the contemporaneous variation in psychological traits through its effects on kinship intensity, independent from Church exposure? Here again, we use the rich database assembled by Schulz et al. [2] on 10 psychological traits observed across countries. Regressing these traits on Distance together with a large set of controls results in Distance being significantly correlated (with the expected signs) with 8 of these psychological traits (Table 3, Panel B). Likewise, Church exposure is significantly correlated with 8 of these traits (Panel A). Including Distance and Church exposure jointly reveals that Church is more frequently significantly correlated with these traits: 6 out of 10, against 4 out of 10 for Distance. About the same conclusion holds when running the analysis on Eurasia, Afro-Eurasia, or the set of countries with little exposure to Western Church (S4 Table in S1 Appendix).

**Table 3:** Country-level regressions of psychological outcomes gon distance to innovations and Church exposure.

	IIP	Indiv.	Creativity	Embeded.	Obedience	Tradition	UN Tickets	Nepotism	Blood Don.	Trust
	Panel A. Church Exposure									
W. Church exp.	0.11***	0.15***	0.12*	-0.13***	-0.56	-0.19***	-0.00	-0.14**	2.99**	0.11*
(in 100 years)	(0.02)	(0.03)	(0.05)	(0.03)	(0.46)	(0.05)	(0.06)	(0.04)	(0.56)	(0.05)
E. Church exp.	-0.04	-0.05	-0.03	0.00	-0.65	0.10	0.14	0.08	0.66	-0.07
(in 100 years)	(0.04)	(0.06)	(0.16)	(0.08)	(0.98)	(0.14)	(0.17)	(0.07)	(1.50)	(0.08)
*N*	147	92	68	68	92	68	137	104	135	67
*R* ^2^	0.583	0.724	0.415	0.713	0.646	0.340	0.274	0.443	0.717	0.394
	**Panel B. Distance**									
Distance	0.17***	0.23**	0.30*	-0.39***	-1.01	-0.43**	-0.07	-0.43***	3.30***	0.38**
(in 1,000 km)	(0.04)	(0.08)	(0.12)	(0.07)	(1.33)	(0.15)	(0.12)	(0.09)	(0.081)	(0.11)
*N*	147	92	68	68	92	68	137	104	135	67
*R* ^2^	0.527	0.640	0.416	0.729	0.645	0.278	0.265	0.451	0.642	0.415
	**Panel C. Distance and Church Exposure**									
Distance	0.08†	0.06	0.20	-0.29***	-0.72	-0.23	-0.06	-0.32**	0.79	0.28*
(in 1,000 km)	(0.05)	(0.08)	(0.14)	(0.07)	(1.55)	(0.18)	(0.14)	(0.10)	(0.69)	(0.14)
W. Church exp.	0.08***	0.14***	0.07	-0.08*	-0.43	-0.14*	0.01	-0.08†	2.80***	0.06
(in 100 years)	(0.02)	(0.04)	(0.07)	(0.03)	(0.52)	(0.07)	(0.07)	(0.05)	(0.61)	(0.05)
E. Church exp.	-0.05	-0.05	-0.04	0.01	-0.64	0.10	0.15	-0.08†	0.57	-0.08
(in 100 years)	(0.05)	(0.06)	(0.17)	(0.08)	(0.98)	(0.15)	(0.17)	(0.05)	(1.53)	(0.07)
*N*	147	92	68	68	92	68	137	104	135	67
*R* ^2^	0.592	0.726	0.437	0.759	0.647	0.371	0.275	0.503	0.718	0.440

This table reports OLS estimates from regressing country-level psychological outcomes on Western and Eastern Church exposure (in 100 years) and ancestry-adjusted distance to innovation (in 1,000 kilometers). Outcome variables are standardized (z-scores). All regressions include the following set of controls: ruggedness, mean distance to waterways, caloric suitability, absolute latitude, and continent fixed effects. Except for distance, data are from Schulz et al. [2]. Robust standard errors are reported in parentheses. VIF values in column 1 of Panel C are 2.10 for Distance, 3.78 for Western Church exposure, and 2.00 for Eastern Church Exposure.

†≤0.1

*≤0.05

**≤0.01

***≤0.001. Significance levels are based on two-tailed tests.

The stronger effect of Church is even clearer when running the analysis on four psychological traits observed at the individual level in Europe and controlling for regions. Church exposure is much more significant, with little effect of Distance (S5 Table in S1 Appendix). On the other hand, focusing on Schulz et al.’s [2] empirical strategy based on second-generation immigrants within Europe (and assigning key variables to mothers’ countries of origins), Distance comes out more powerful than Church in explaining the same four psychological traits (Table 4). These last results are especially interesting as this empirical strategy enables to isolate cultural traits that are transmitted within families while controlling for unobservable characteristics of countries in which individuals grew up. Overall, explaining the same psychological traits, Distance is unrelated when respondents are from European ascent, but strongly significant when analyzing responses by children of immigrants. As we capture descendants of immigrants from all over the world, these results suggest that Distance plays a key role outside Europe in explaining psychological traits. On the other hand, Church has a specific effect on European populations.

**Table 4:** Individual-level regressions of psychological outcomes on distance to innovations and Church exposure.

	IIP	Obedience	Trust	Fairness
	Panel A. Church Exposure			
W. Church exp.	0.024*	-0.029**	0.020**	0.016*
(in 100 years)	(0.007)	(0.010)	(0.006)	(0.006)
E. Church exp.	0.035**	-0.053***	0.025†	0.016*
(in 100 years)	(0.012)	(0.012)	(0.015)	(0.006)
*N*	13,389	13,389	14,627	14,567
*R* ^2^	0.058	0.127	0.092	0.077
	**Panel B. Distance**			
Distance	0.050*	-0.103**	0.058***	0.057***
(in 1,000 km)	(0.023)	(0.030)	(0.013)	(0.007)
*N*	13,389	13,389	14,627	14,567
*R* ^2^	0.057	0.129	0.092	0.077
	**Panel C. Distance and Church Exposure**			
Distance	0.024	-0.089**	0.042***	0.045***
(in 1,000 km)	(0.025)	(0.027)	(0.12)	(0.013)
W. Church exp.	0.019*	-0.012	0.012†	0.007
(in 100 years)	(0.007)	(0.009)	(0.006)	(0.007)
E. Church exp.	0.031*	-0.039***	0.019	-0.005
(in 100 years)	(0.012)	(0.010)	(0.014)	(0.0011)
*N*	13,389	13,389	14,627	14,567
*R* ^2^	0.058	0.129	0.093	0.078

This table reports OLS estimates from regressing individual-level ESS-based psychological outcomes on Western Church exposure (in 100 years) and ancetry-adjusted distance to innovations (in 1,000 kilometers). Outcome variables are standardized (z-scores). All regressions include the following set of controls: country and ESS wave fixed effects, gender, age, and age squared, agricultural suitability, absolute latitude, mean distance to the sea, and average terrain ruggedness. Except for distance, data are from Schulz et al. [2]. Robust standard errors clustered for the 440 regions are reported in parentheses.

†≤0.1

*≤0.05

**≤0.01

***≤0.001. Significance levels are based on two-tailed tests.

### Diffusion or direct effect of the presence of agriculture?

A mechanism close to the one we study might well be that it is the presence of agriculture *per se* that led to family complexification over time, potentially through population growth and the implied competition for land. Other innovations could indeed be the result of a direct effect of agriculture. Borcan et al. [25] focus on the causal effect of the adoption of agriculture to explain the emergence of the state, which was independently invented in six different innovation centers [26]. They further control for a potential diffusion mechanism from these six pristine states, assuming specific areas of diffusion (e.g., both Gabon and Venezuela are in the Western diffusion area). Scott [27] also assumes that social complexity in general is the product of agriculture, especially storable grains. In contrast, Johnson and Earle [28] reject a pure effect of the presence of agriculture as “farmers could be observed at any level of social complexification,” stressing population density as the leading factor.

In the Toddian framework we adopt, agriculture is initially necessary to observe innovations in family systems because high population densities stimulate family complexification. However, once invented, family innovations disseminate without population constraints and irrespective of the timing of the development of agriculture. For instance, the Nomads of Eurasian steps—who did not suffer any constraint on land—complexified their family organization through their interactions with populations that were already complexified in the middle East [3: 142–6]. Such a diffusion process therefore makes it possible to observe complex families absent of farming, sedentary lifeways, or land scarcity. For instance, farmers in current Ukraine exhibited complex families in the nineteenth century despite abundant land [3: 318–9]. This model of diffusion is akin to that of writing, which was invented in a few centers (Mesopotamia, perhaps Egypt, China and Meso-America) before spreading to other areas regardless the timing of the transition to agriculture [29]. A latter innovation and crucial family behavior, fertility control, also spread through diffusion according to [30].

Unfortunately, it remains challenging to empirically disentangle our diffusion hypothesis from that of the presence of agriculture *per se*. To explore this possibility, we use the date of agriculture adoption from [31] and the same migration matrix to build ancestry-adjusted values of transition to agriculture (henceforth Timing). Timing and Distance are seldom correlated when unconditionally compared (*ρ* = −0.01), though they are more so within continents (*ρ* = −0.32), as in each diffusion area, the shorter is the distance, the earlier the adoption of agriculture. To offer a first view of these competing mechanisms, we reproduce the cross-country analysis of Table 1 but with Timing instead of Church exposure (S6 Table in S1 Appendix). As Distance, Timing is also significantly correlated with the distribution of the KII and cousin marriage rates across countries: the longer the presence of agriculture, the higher the kinship intensity. While a naïve observer would associate nuclear families with modernity due to their joint presence in the West, the correlation between Timing and kinship intensity supports the idea that complex family organizations constitute an evolved form of organization rather than an initial one. A broad view of long history can dismantle this naïve view as European populations long lagged behind in terms urbanization, writing and statehood. Our framework also assumes kinship intensity as the product of a long history but through diffusion of innovations rather than a mechanical implication of agriculture adoption.

To try to disentangle whether the complexification of family organizations is a mechanical implication of the presence of agriculture rather than the result of the reception of innovations from centers that spread both agriculture and family innovations, as we argue, we include Distance and Timing together in the regression analysis (S6 Table in S1 Appendix). While both variables help explain the KII and cousin marriage rates worldwide and across Eurasia, only Timing is strongly significant on the sample of countries with limited Church exposure. While jointly included, Timing is the only significant variable on almost all subsamples in explaining both KII and cousin marriage rates. In contrast, Distance is more frequently significant when relaxing continent fixed effects, a setting in which Distance and Timing exhibit little correlation (S7 Table in S1 Appendix). These unclear results suggest that the three-way relationship between Distance to innovations, Timing of transition to agriculture, and kinship intensity is complex and constitutes a fruitful area for further research.

Still, to explore more deeply the potential direct effects of the appearance of agriculture relative to the role of diffusion, we reproduce Table 3 (explaining various psychological outcomes across countries) when replacing Church exposure with Timing (S8 Table in S1 Appendix). This last analysis shows that Timing is never significant in explaining the psychological outcomes when taken together with Distance, which show that time since agriculture is not an effective mechanism affecting psychological outcomes across countries relative to Distance to innovations. Lastly, we must note that it is not possible to explore the effect of Timing at the regional level because data on agriculture appearance is only available at a country scale, contrary to our measure of Distance.

## Discussion

Overall, our analyzes imply that Distance to innovations explains kinship intensity across countries and within European countries better than Church exposure does. Still, we cannot exclude the possibility of a direct effect of the presence of agriculture, although this explanation seems unrelated to the current distribution of psychological traits once diffusion is accounted for. In contrast, Church exposure remains better at explaining psychological outcomes in Europe, though less clearly so across the globe. This suggests that while Distance to innovations is the most plausible explanation of kinship intensity, the Church’s MFP, even if rooted in Distance, could have played an additional role, potentially reinforcing pre-existing psychological traits especially in Europe.

Our approach of family organization as the fundamental cause explains why the MFP was developed by religious authorities in Western Europe only, while Schulz et al. [2] approach is silent on the origin of the MFP. Indeed, the MFP has no equivalent in other religious areas and is not a consequence of the contents of the New Testament as other forms of Christianity, especially Eastern Christians, did not promote the MFP. Among the different human activities that are affected by variations in psychological traits, Schulz et al. [2] suggest that WEIRD psychologies sustain important cooperation of individuals free of kin-links especially the development of representative institutions. The prevalence of the nuclear family is indeed a plausible condition of the emergence of this kind of institutions. The existence of numerous antic representative institutions (the Roman republic, *civitates*, …) in Western Europe thus implied the prevalence of the nuclear family at this time. This is inexplicable by Church exposure while consistent with our framework of our age-old local low kinship intensity in Western Europe.

## Supporting information

S1 Appendix (PDF)
