# Supplementary material for: Distance to innovations, kinship intensity, and psychological traits
Source: PLoS One. 2023 Jan 26;18(1):e0279864. doi: 10.1371/journal.pone.0279864 (PMC9879433; doi:10.1371/journal.pone.0279864)
Supplement: S1 Appendix — (PDF) [file pone.0279864.s001.pdf]

Supporting Information for

Distance to Innovations, Kinship Intensity, and Psychological Traits

David le Bris Victor Gay

December 2022

This PDF file includes:

The prevalence of the nuclear family before 1000 CE in Western Europe.

Tables S1 to S8.

Figure S1.

The prevalence of the nuclear family before 1000 CE in Western Europe

Studying the last hunter-gatherers populations worldwide, anthropologists have found evidence of a nuclear family organization among many. The nuclear family is observed for instance in Africa among the Kung! of the Kalahari (Johnson and Earle, 2000), the Agta in the Philippines (Headland, 1987), the Ona and the Yaghan of Tierra del fuego (Stuart, 1972), and the Shoshone in North America (Johnson and Earle, 2000). Apart from certain phases of gathering or hunting, these populations live in small bands grouping up to a few dozens of nuclear families. Beyond the nuclear family, the flexibility of social relations is the rule (Lee and Devore, 1968) without systematic kin relationships. These mobile groups are composed along variable criteria that always leave room for opportunities and choices, in particular a choice between paternal and maternal relations. It is frequently assumed that these populations reflect the family organization of the homo sapiens that have been practiced for millenniums.

Evidence from the period that follows the Neolithic revolution remains scarce but generally supports the notion of some persistence of the nuclear family that characterizes hunter-gatherer populations, although some archaeological evidence from the Near East and Mesoamerica suggests that family complexification occurred quickly along with the agricultural revolution (Flannery, 2002). While such a complexification is consistent with our framework, we posit that agriculture is not mechanically associated with complex families as farmers could be observed at any level of social complexification (Johnson and Earle, 2000). Indeed, collective tombs in Germanic areas are consistent with the prevalence of nuclear families: never more than two adults, a high frequency of adult and children, and most of the time, less than four individuals in each tomb (Falkenstein, 2005). Several human remains have been deeply analyzed using DNA and Isotopic approaches. Studying the burials after a raid in Saxony-Anhalt, Haak et al. (2008) claim to "have established the presence of the classic nuclear family in a prehistoric context in Central Europe." Skeletons from early Neolithic South Germany are "consistent with the potential identification of Neolithic nuclear families" (Bentley, 2003). A burial in the Pyrenees "suggests the possibility of a traditional nuclear family" during the Neolithic time (Gomes et al., 2020). Analyzing two South Germany cemeteries from late Copper Age Bell Beaker culture, Sjögren et al. (2020) conclude that "The basic kinship units are nuclear families." Most of these studies support patrilocality of these nuclear families, suggesting an entry level of kinship intensity. Conversely, the very high female genetic diversity, implying female exogamy, excludes high rates of of cousin marriage characteristic of a strong kinship intensity. Interestingly, patrilocality associated with female exogamy is typical of the stem family observed in modern Germany, in which the

eldest son remains at his parental home, marrying a non-kin wife. Otherwise, in Southern France, several villages in dry stones of the Fontbouisse culture have been fully preserved, exhibiting houses which sizes are consistent with the nuclear family (Guilaine, 2016).

More evidence is available from the Antiquity. There is a broad interdisciplinary consensus to accept that the Roman family, even if characterized by patrilineal orientation—especially among the elite—was nuclear. This conclusion has been clear for long according to the Roman law. "The Roman family seems to have been a 'nuclear family' like our own" (Crook, 1967, 98) and is now widely accepted in other fields. For instance: "classical scholars now tend to assume that the chief Roman residential unit was the nuclear family" (Dixon, 1988, 9); "most scholars now agree that the Romans lived in some kind of nuclear families" (Lassen, 1997, 116); "It has become increasingly clear over recent decades that the nuclear family structure was the norm among Roman citizens in the classical period, at least in the western half of the empire" (Parkin and Pomeroy, 2007, 74).

We list some of the evidence. Monogamy was undoubtedly the rule even for the elite. Family representations on funerary reliefs are dominated by the couple and children (George, 2005, 37). Saller (1994) provides various forms of evidence, especially from the Roman literature supporting the prevalence of the nuclear family. According to the number of houses and population of Pompeii and Herculanum, the average household size was between 6.7 and 8.3 (Wallace-Hadrill, 1994, 99), which is not inconsistent with a nuclear family in a context of domestic slavery; there is however a large variation in house size reflecting economic inequalities.

Numerous evidence has been provided by the analysis of funeral epigraphy. Shaw and Saller (1984) highlight that 88% of Western inscriptions were related to the nuclear family. Martin (1996) criticized their method and observe a share of only 59% on an Asia minor sample. Applying the two methods to epigraphic from Lusitania, Edmondson (2005) shows that in both cases the nuclear family is the most present (about 85%). One can doubt the reliability of these statistics to perfectly reflect the regular family organization, but the large majority of these inscriptions is related to the nuclear family (Huebner, 2010).

Roman law of inheritance organized the transmission to offspring only, sharing assets among all siblings (boys and girls). This is true from the first texts (Twelve tables), in the Theodosian Code, in the famous Justinian Code (Novelle 118 and 127), and finally in the Alaric breviary issued by Visigoths to rule the Gallo-roman populations. And in case of the lack of children, all the assets were transmitted to the closest parent only and not shared among collaterals. Moreover, the freedom to bequest was always present. The Justinian Code was applied in part of Europe until the 18th century and, regarding inheritance, reproduced in the French Code Civil. Such transmission rules cannot sustain a high kinship intensity

family. In a framework of intensive kinship, inheritance rules should reflect these links, as in the Sharia law that organized inheritance to different collaterals or keep common the assets.

Cousin marriage is only known for few exceptional cases in dominant families (similar to what has been observed among European royal families until the 20th century). Among the Roman patricians, on 33 identified marriages before the 3rd century, none was endogamic (Shaw and Saller, 1984). But the best evidence of the very low prevalence of cousin marriage at the end of Roman times is from St Augustine (The City of God, XV, 16), written between 413 and 426. He mentions that, even if not forbidden, marriage with cousin were very rare and "disgusting":

> And with regard to marriage in the next degree of consanguinity, marriage between cousins, we have observed that in our own time the customary morality has prevented this from being frequent, though the law allows it. It was not prohibited by divine law, nor as yet had human law prohibited it; nevertheless, though legitimate, people shrank from it, because it lay so close to what was illegitimate, and in marrying a cousin seemed almost to marry a sister—for cousins are so closely related that they are called brothers and sisters, and are almost really so.

To capture the strength of kinship ties, Schultz et al. (2019) retain five characteristics building their Kinship Intensity Index that would all score weakly in the Roman time: *cousin marriage* (socially banned), *polygamy* (legally forbidden), *co-residence of extended family* (sometimes observed but only among the elite), *lineage organization* (declining role of the *pater familias* after the Republic and cognate kinship always recognized) and *community organization* (a Roman didn't live in a specific lineage' place and married freely outside locals).

After the fall of the Roman Empire, we have to distinguish the behavior of the local population from that of the few Germanic rulers. This distinction is supported by paleogenomics showing different patterns between invaders and local populations (Amorim et al., 2018). Originating in regions closer to innovations centers, Germanic populations have been exposed to more complexification than Western Europe (Todd, 2011) even if the induced kinship intensity remained weak in a worldwide comparison.

In these ruling elites, the nuclear family, even if included in a bilateral network of parents (the sippen), was dominant. It is important to note that only patrilinearity characterizes an increase of the kinship intensity affiliating the couple to a single filiation. When the couple interacts with both sides (bilaterality), as in modern western case, the kinship intensity remains very low because the couple have always the option to privilege one side or the

other (see, SBBH S1.1.4). “Nobody, it seems, defend today that the society of barbarian kingdom were based on a clanic or tribal organization” (Guichard, 1986). For the Visigoths, King (1972, 222–3) concludes his study “Kinship as a group possessed very little power, and whatever power it had, it was subordinate to that of the parents (father and mother) anyway. It was the monogamous family that now formed the basic social unit.” Murray (1983) devoted a whole book to contest that Germanic populations, even before their entrance into the Roman world, were clan-based: “These studies show that there is no evidence for the idea that the society of the ancient Germans was rooted in a clan or extensive lineage structure” (Murray, 1983, 8). These analyses are based on customs, laws and other form of evidence. For instance, in Alemannic Switzerland, studying customs and gifts to Saint-Gall abbey, for which archives have been conserved from 724, Goezt concludes that, as early as the 5th century, inheritance rules and practices among the elites favored siblings rather than collaterals supporting the nuclear family (Goetz, 2005, 235). The primogeniture for noble fief (transferring the main asset to the eldest son), characterizing an increase in the kinship intensity, appeared around year 1000; before this innovation, assets, including Kingdoms were divided among sons. In Frankish aristocracy, each spouse owned their personal assets even if managed by husbands, and children left the parental home upon marriage or earlier. “Whether one places oneself upstream or downstream from the Carolingian period, we must therefore renounce the myth of the big house housing a large, polynuclear, patriarchal-type family” (Le Jan, 1995, 455).

For local populations, as early as archives are available, households were consistent with nuclear family. On the properties of the Saint-Germain-des-Prés in the Paris area around 820, the number of inhabitants of each household was about five (Coleman, 1971) with only eight household with more than nine members. The same five figure was observed from documents of Saint-Victor of Marseille (Zerner, 1981), in Abbruzes through San Vincenzo al Volturno documents (Wickham, 1982) and in the Benevent in 770 using Monte Cassino Abbey archives. Between 5 and 6 is also documented in Rhenany in 804–886 (Kuchenbuch, 1978). According to the last thirty years of French archeological research, the average size of rural houses of these times was around 70 m^2 which here again supports the nuclear family (Peytremann, 2003). Analyzing the life chronicles (*Vitae*) of the saints of the Merovingian times (481–751), Theis (1976) identified only nuclear families in which children leave the parental household for marriage. So, as observed in the 8th–9th century, “the conjugal family was the single fundamental structure” on the continent (Toubert, 1976, 711). The Old English provides some information for the Anglo-Saxons. Terms for lineal ascendants were based on those for the nuclear family supporting Ghurye (1955, 7, 13) to see the primacy, even in Anglo-Saxon times, of the nuclear family as against any wider kin group.

"The complete lack of specificity in terms for cousins of various degrees, which would be all-important in the operation of a wide-ranging bilateral system, suggests that these kin and the distinctions between them was not regularly of major significance" (Lancaster, 1958). According to the same author, a man was called a *bonda* leading to the word *hus-bonda* (man of the house) to refer to a husband which again suggest the importance of the nuclear household.

Finally, cousin marriage remained as infrequent as when depicted by Saint Augustine, even for nobility. "Examining the various types of sources, myths founders, legends, historical accounts, anthroponymy or genealogies, lastly the lives of saints, shows that the Germans were indeed fundamental exogamous" (Le Jan, 1995, 409). According to the available genealogies under the Merovingians, intermarriage between Frankish and Burgunds, or with local Gallo-romans were frequent excluding by nature cousin marriage. Another evidence is the frequency of family names build as -gund in the Frankishes families close to Burgundy implying also a cross marriage (Le Jan, 1995, 410). After the Anglo-saxon conquest of England, marriages were also always exogamic among nobility according to the available data (Fisher, 1973, 118–9).

From the Neolithic to the Middle Ages, the nuclear family have thus been prevalent in Western Europe even if the presence of more complex organization is occasionally documented. This picture is very close to the 19th century one, during which the nuclear form dominated unchallenged in the northwest Europe while more complex organizations were observed in some areas (see, e.g., Duranton, Rodríguez-Pose and Sandall, 2009); for instance the stem family in the Germanic zone and around the Pyrenees and even the more complex exogamic communitarian family in central Italy (about the former Papal states and Tuscany), which is highly inconsistent with SBBH's view of Rome as the source of the nuclear family in Europe.

Table S1 — Standardized Cross-Country Regressions of KII and Cousin Marriage on Distance to Innovations and Church Exposure

	Panel A. Kinship Intensity Index (KII)											
Sample:	World			Eurasia			Afro-Eurasia			W. Church exp. < 50 years		
Distance		-0.38***	-0.34***		-0.72***	-0.77***		-0.31***	-0.28***		-0.17†	-0.15†
(in 1,000 km)		(0.07)	(0.07)		(0.10)	(0.13)		(0.07)	(0.07)		(0.09)	(0.09)
W. Church exp.	-0.31***		-0.11	-0.20†		0.06	-0.20*		-0.10	-2.29		-1.27
(in 100 years)	(0.08)		(0.08)	(0.10)		(0.09)	(0.10)		(0.09)	(2.64)		(3.05)
E. Church exp.	-0.07		-0.03	-0.01		-0.01	-0.02		-0.01	-0.08		-0.06
(in 100 years)	(0.08)		(0.07)	(0.10)		(0.09)	(0.09)		(0.08)	(0.11)		(0.11)
N	147	147	147	74	74	74	118	118	118	93	93	93
R^2	0.615	0.667	0.670	0.483	0.642	0.645	0.609	0.648	0.652	0.485	0.499	0.503
	**Panel B. Log Rate of Cousin Marriage**											
Sample:	World			Eurasia			Afro-Eurasia			W. Church exp. < 50 years		
Distance		-0.29*	-0.26*		-0.42*	-0.40*		-0.36**	-0.34**		-0.29†	-0.31†
(in 1,000 km)		(0.12)	(0.11)		(0.21)	(0.19)		(0.12)	(0.10)		(0.16)	(0.18)
W. Church exp.	-0.21		-0.10	-0.25		-0.20	-0.27		-0.21	-0.30		1.88
(in 100 years)	(0.15)		(0.14)	(0.20)		(0.17)	(0.19)		(0.17)	(2.44)		(2.71)
E. Church exp.	0.63		0.18	0.62		-0.53	0.56		-0.59	1.07		0.98
(in 100 years)	(2.14)		(2.13)	(2.24)		(2.02)	(2.20)		(1.99)	(2.96)		(2.30)
N	69	69	69	41	41	41	51	51	51	33	33	33
R^2	0.742	0.766	0.768	0.742	0.773	0.783	0.754	0.786	0.795	0.260	0.338	0.346

This table reports standardized OLS estimates from regressing country-level kinship intensity indexes (KII) in Panel A and country-level rates of cousin marriage (in log) in Panel B on Western and Eastern Church exposure (in 100 years) and ancestry-adjusted distance to innovation (in 1,000 kilometers). All regressions include the following set of controls: ruggedness, mean distance to waterways, caloric suitability, absolute latitude, and continent fixed effects. Except for distance, data are from SBBH. Robust standard errors are reported in parentheses. For the World sample, VIF values when variables are jointly included are 2.10 for Distance, 3.78 for Western Church exposure, and 2.00 for Eastern Church exposure in Panel A; 3.15 for Distance, 5.69 for Western Church exposure, and 1.63 for Eastern Church exposure in Panel B.

†≤ 0.1, * P ≤ 0.05, ** P ≤ 0.01, *** P ≤ 0.001. Significance levels are based on two-tailed tests.

Table S2 — Cross-Country Regressions of KII and Cousin Marriage on Minimum Distance to Innovations and Church Exposure

	Panel A. Kinship Intensity Index (KII)											
Sample:	World			Eurasia			Afro-Eurasia			W. Church exp. < 50 years		
Minimum Distance		-0.21***	-0.17***		-0.40***	-0.44***		-0.18***	-0.16**		-0.06	-0.05
(in 1,000 km)		(0.04)	(0.05)		(0.08)	(0.09)		(0.05)	(0.05)		(0.06)	(0.06)
W. Church exp.	-0.12***		-0.09**	-0.07†		-0.04	-0.07*		-0.06†	-0.86		-0.69
(in 100 years)	(0.03)		(0.03)	(0.04)		(0.03)	(0.04)		(0.03)	(0.99)		(1.08)
E. Church exp.	-0.06		-0.08	-0.01		-0.10	-0.01		-0.04	-0.07		-0.07
(in 100 years)	(0.06)		(0.06)	(0.08)		(0.08)	(0.08)		(0.07)	(0.10)		(0.09)
N	147	147	147	74	74	74	118	118	118	93	93	93
R^2	0.615	0.623	0.640	0.483	0.580	0.591	0.609	0.630	0.638	0.485	0.482	0.489
	Panel B. Log Rate of Cousin Marriage											
Sample:	World			Eurasia			Afro-Eurasia			W. Church exp. < 50 years		
Minimum Distance		-0.28†	-0.23†		-0.32	-0.28		-0.34*	-0.32*		-0.06	-0.06
(in 1,000 km)		(0.14)	(0.12)		(0.23)	(0.18)		(0.15)	(0.13)		(0.18)	(0.19)
W. Church exp.	-0.15		-0.11	-0.17		-0.16	-0.19		-0.18	-0.21		0.09
(in 100 years)	(0.10)		(0.10)	(0.14)		(0.12)	(0.13)		(0.11)	(1.69)		(1.65)
E. Church exp.	0.99		0.22	0.97		-0.41	0.88		-0.78	1.67		1.61
(in 100 years)	(3.35)		(3.36)	(3.50)		(3.62)	(3.43)		(3.39)	(4.62)		(4.67)
N	69	69	69	41	41	41	51	51	51	33	33	33
R^2	0.742	0.747	0.754	0.742	0.739	0.754	0.754	0.760	0.775	0.260	0.256	0.261

This table reports OLS estimates from regressing country-level kinship intensity indexes (KII) in Panel A and country-level rates of cousin marriage (in log) in Panel B on Western and Eastern Church exposure (in 100 years) and ancestry-adjusted distance to innovation (in 1,000 kilometers). All regressions include the following set of controls: ruggedness, mean distance to waterways, caloric suitability, absolute latitude, and continent fixed effects. Except for distance, data are from SBBH. Robust standard errors are reported in parentheses. For the World sample, VIF values when variables are jointly included are 1.47 for Distance, 3.30 for Western Church exposure, and 1.98 for Eastern Church exposure in Panel A; 1.79 for Distance, 5.41 for Western Church exposure, and 1.69 for Eastern Church exposure in Panel B.

†$\leq$ 0.1, * P $\leq$ 0.05, ** P $\leq$ 0.01, *** P $\leq$ 0.001. Significance levels are based on two-tailed tests.

Table S3 — Standardized Region-Level Regressions of Cousin Marriage on Distance to Innovation and Church Exposure

	Log Rate of Cousin Marriage				
Distance		-0.352***	-0.256**	-0.343***	-0.383*
(in 1,000 km)		(0.062)	(0.092)	(0.085)	(0.182)
W. Church exp.	-0.454***		-0.187	0.000	0.120
(in 100 years)	(0.101)		(0.124)	(0.116)	(0.094)
Caroligian Empire				-0.317***	-0.200**
				(0.090)	(0.067)
N	68	68	68	68	68
R^2	0.900	0.910	0.914	0.930	0.947
Geographic controls	Yes	Yes	Yes	Yes	Yes
Country FE	No	No	No	No	Yes

This table reports OLS estimates from regressing region-level rates of cousin marriage (in log) on Western Church exposure (in 100 years), exposure to the Caroligian Empire, and ancestry-adjusted distance to innovation (in 1,000 kilometers). Included are regions of Spain, France, Italy, and Turkey. All regressions include the following set of controls: terrain ruggedness, distance to the coast, caloric suitability, absolute latitude, precipitation, temperature, elevation, presence of river or lake, irrigation potential, caloric suitability for oats and for rye. Except for distance, data are from SBBH. Robust standard errors are reported in parentheses. †$\leq$ 0.1, * P $\leq$ 0.05, ** P $\leq$ 0.01, *** P $\leq$ 0.001. Significance levels are based on two-tailed tests.

Table S4 — Country-Level Regressions of Psychological Outcomes on Distance to Innovations and Church Exposure

	IIP	Individ.	Creativity	Embedded.	Obedience	Tradition	UN Tickets	Nepotism	Blood Don.	Trust
Panel A. Eurasia										
Distance	0.16*	0.11†	0.03	0.12	0.10	-0.05	0.11	0.22	0.05	-0.41***
(in 1,000 km)	(0.07)	(0.06)	(0.06)	(0.11)	(0.09)	(0.11)	(0.18)	(0.16)	(0.17)	(0.11)
W. Church exp.	0.07***	0.08***	0.76	0.12**	0.12**	2.00*	0.05	0.05	0.74	-0.09*
(in 100 years)	(0.02)	(0.02)	(0.80)	(0.04)	(0.04)	(0.95)	(0.07)	(0.08)	(1.63)	(0.04)
E. Church exp.	-0.02	-0.05	0.01	-0.06	-0.07	-0.03	-0.01	0.05	0.09	-0.04
(in 100 years)	(0.05)	(0.05)	(0.05)	(0.06)	(0.06)	(0.05)	(0.13)	(0.08)	(0.16)	(0.09)
N	74	118	93	55	71	43	42	55	41	46
R^2	0.682	0.606	0.188	0.684	0.696	0.324	0.522	0.384	0.426	0.727
Panel B. Afro-Eurasia										
Distance	-0.28*	-0.24†	-3.42	-0.77	-0.21	-0.21	-0.28	-0.36	-0.31	-0.09***
(in 1,000 km)	(0.07)	(0.11)	(2.12)	(1.58)	(2.86)	(0.18)	(0.18)	(0.26)	(0.27)	(0.15)
W. Church exp.	-0.10***	5.61***	-0.10	-0.42**	0.78**	-0.23*	-0.22	4.18	0.03	0.01*
(in 100 years)	(0.04)	(2.32)	(0.60)	(0.54)	(18.68)	(0.08)	(0.07)	(2.08)	(0.09)	(0.08)
E. Church exp.	-0.02	-0.10	-0.46	-0.66	-1.52	-0.07	-0.00	4.18	0.06	0.13
(in 100 years)	(0.08)	(0.09)	(1.10)	(1.00)	(1.21)	(0.16)	(0.16)	(2.08)	(0.20)	(0.19)
N	55	29	60	75	48	42	55	41	68	111
R^2	0.771	0.826	0.447	0.647	0.621	0.515	0.431	0.431	0.215	0.236
Panel C. W. Church exp. < 50 years										
Distance	-0.01*	-0.46†	-0.35	-0.21	2.49	0.67	0.03	0.15	0.32	0.03***
(in 1,000 km)	(0.18)	(0.17)	(0.11)	(0.16)	(2.33)	(0.79)	(1.05)	(0.18)	(0.14)	(0.15)
W. Church exp.	1.13***	-0.04***	-0.06	1.58**	2.50**	2.74*	4.29	0.10	0.07	-0.01*
(in 100 years)	(2.38)	(0.05)	(0.05)	(1.46)	(0.78)	(0.67)	(13.29)	(0.05)	(0.06)	(1.68)
E. Church exp.	0.11	0.10	0.12	0.04	0.76	0.46	2.00	0.10	-0.05	0.09
(in 100 years)	(0.24)	(0.06)	(0.07)	(0.07)	(1.67)	(1.54)	(1.93)	(0.05)	(0.08)	(0.08)
N	87	58	79	52	64	107	83	42	55	40
R^2	0.152	0.637	0.527	0.247	0.620	0.733	0.355	0.537	0.414	0.379

This table reports OLS estimates from regressing country-level psychological outcomes on Western and Eastern Church exposure (in 100 years) and ancestry-adjusted distance to innovation (in 1,000 kilometers). Outcome variables are standardized (z-scores). All regressions include the following set of controls: ruggedness, mean distance to waterways, caloric suitability, absolute latitude, and continent fixed effects. Except for distance, data are from SBBH. Robust standard errors are reported in parentheses.

†≤ 0.1, * P ≤ 0.05, ** P ≤ 0.01, *** P ≤ 0.001. Significance levels are based on two-tailed tests.

Table S5 — Individual-Level Regressions of Psychological Outcomes on Distance to Innovations and Church Exposure Across Western European Regions

	IIP	Obedience	Trust	Fairness
**Panel A. Church exposure**				
Church exposure	0.009*	-0.015**	0.012***	0.012***
(in 100 years)	(0.005)	(0.005)	(0.004)	(0.003)
N	208,587	208,587	228,844	227,388
R^2	0.069	0.213	0.147	0.142
**Panel B. Distance**				
Distance	0.032	-0.006	0.015	0.009
(in 1,000 km)	(0.031)	(0.043)	(0.037)	(0.031)
N	208,587	208,587	228,844	227,388
R^2	0.069	0.212	0.146	0.142
**Panel C. Distance and Church Exposure**				
Distance	0.021	0.014	-0.002	-0.008
(in 1,000 km)	(0.031)	(0.040)	(0.034)	(0.030)
Church exposure	0.009†	-0.015**	0.013***	0.012***
(in 100 years)	(0.005)	(0.006)	(0.004)	(0.003)
N	208,587	208,587	228,844	227,388
R^2	0.069	0.213	0.147	0.142

This table reports OLS estimates from regressing individual-level ESS-based psychological outcomes on Western Church exposure (in 100 years) and ancetry-adjusted distance to innovations (in 1,000 kilometers). Outcome variables are standardized (z-scores). All regressions include the following set of controls: country and ESS wave fixed effects, gender, age, and age squared, agricultural suitability, absolute latitude, mean distance to the sea, and average terrain ruggedness. Except for distance, data are from SBBH. Robust standard errors clustered for the 440 regions are reported in parentheses.

†≤ 0.1, * $P \leq 0.05$, ** $P \leq 0.01$, *** $P \leq 0.001$. Significance levels are based on two-tailed tests.

Table S6 — Cross-Country Regressions of KII and Cousin Marriage on Distance to Innovations and Time since Transition to Agriculture

	Panel A. Kinship Intensity Index (KII)											
Sample:	World			Eurasia			Afro-Eurasia			W. Church exp. < 50 years		
Distance	-0.46*		-0.15	-0.67*		-0.20	-0.57**		-0.21	-0.47†		-0.19
(in 1,000 km)	(0.18)		(0.17)	(0.33)		(0.35)	(0.19)		(0.22)	(0.25)		(0.25)
Timing		0.38***	0.33**		0.39**	0.32*		0.36**	0.28*		0.27*	0.21
(in 1,000 years)		(0.10)	(0.10)		(0.13)	(0.14)		(0.11)	(0.13)		(0.12)	(0.14)
N	69	69	69	41	41	41	51	51	51	33	33	33
R^2	0.766	0.800	0.803	0.773	0.800	0.803	0.786	0.804	0.808	0.338	0.367	0.375
	Panel B. Log Rate of Cousin Marriage											
Sample:	World			Eurasia			Afro-Eurasia			W. Church exp. < 50 years		
Distance	-0.46*		-0.15	-0.67*		-0.20	-0.57**		-0.21	-0.47†		-0.19
(in 1,000 km)	(0.18)		(0.17)	(0.33)		(0.35)	(0.19)		(0.22)	(0.25)		(0.25)
Timing		0.38***	0.33**		0.39**	0.32*		0.36**	0.28*		0.27*	0.21
(in 1,000 years)		(0.10)	(0.10)		(0.13)	(0.14)		(0.11)	(0.13)		(0.12)	(0.14)
N	69	69	69	41	41	41	51	51	51	33	33	33
R^2	0.766	0.800	0.803	0.773	0.800	0.803	0.786	0.804	0.808	0.338	0.367	0.375

This table reports OLS estimates from regressing country-level kinship intensity indexes (KII) in Panel A and country-level rates of cousin marriage (in log) in Panel B on ancestry-adjusted distance to innovation (in 1,000 kilometers) and timing since agriculture (in 1,000 years). All regressions include the following set of controls: ruggedness, mean distance to waterways, caloric suitability, absolute latitude, and continent fixed effects. Except for distance and timing, data are from SBBH. Robust standard errors are reported in parentheses. For the World sample, VIF values when variables are jointly included are 1.88 for Distance and 2.85 for Timing in Panel A; 3.99 for Distance and 3.02 for Timing in Panel B.
†≤ 0.1, * P ≤ 0.05, ** P ≤ 0.01, *** P ≤ 0.001. Significance levels are based on two-tailed tests.

Table S7 — Cross-Country Regressions of KII and Cousin Marriage on Distance to Innovations and Time since Transition to Agriculture Without Continent Fixed Effects

	Panel A. Kinship Intensity Index (KII)											
Sample:	World			Eurasia			Afro-Eurasia			W. Church exp. < 50 years		
Distance	-0.84***		-0.75***	-0.78**		-0.38	-0.60***		-0.51**	-0.42*		-0.44*
(in 1,000 km)	(0.14)		(0.14)	(0.25)		(0.31)	(0.16)		(0.16)	(0.20)		(0.21)
Timing		0.32***	0.20**		0.44***	0.30†		0.22**	0.15*		0.08	0.10
(in 1,000 years)		(0.09)	(0.07)		(0.12)	(0.16)		(0.08)	(0.07)		(0.07)	(0.07)
N	69	69	69	41	41	41	51	51	51	33	33	33
R^2	0.646	0.527	0.678	0.768	0.782	0.793	0.773	0.736	0.791	0.322	0.218	0.349
	Panel B. Log Rate of Cousin Marriage											
Sample:	World			Eurasia			Afro-Eurasia			W. Church exp. < 50 years		
Distance	-0.84***		-0.75***	-0.78**		-0.38	-0.60***		-0.51**	-0.42*		-0.44*
(in 1,000 km)	(0.14)		(0.14)	(0.25)		(0.31)	(0.16)		(0.16)	(0.20)		(0.21)
Timing		0.32***	0.20**		0.44***	0.30†		0.22**	0.15*		0.08	0.10
(in 1,000 years)		(0.09)	(0.07)		(0.12)	(0.16)		(0.08)	(0.07)		(0.07)	(0.07)
N	69	69	69	41	41	41	51	51	51	33	33	33
R^2	0.646	0.527	0.678	0.768	0.782	0.793	0.773	0.736	0.791	0.322	0.218	0.349

This table reports OLS estimates from regressing country-level kinship intensity indexes (KII) in Panel A and country-level rates of cousin marriage (in log) in Panel B on ancestry-adjusted distance to innovation (in 1,000 kilometers) and timing since agriculture (in 1,000 years). All regressions include the following set of controls: ruggedness, mean distance to waterways, caloric suitability, and absolute latitude. Except for distance and timing, data are from SBBH. Robust standard errors are reported in parentheses. For the World sample, VIF values when variables are jointly included are 1.65 for Distance and 1.61 for Timing in Panel A; 1.77 for Distance and 1.38 for Timing in Panel B.
†≤ 0.1, * P ≤ 0.05, ** P ≤ 0.01, *** P ≤ 0.001. Significance levels are based on two-tailed tests.

Table S8 — Country-Level Regressions of Psychological Outcomes on Distance to Innovations and Timing of Agriculture

	IIP	Individ.	Creativity	Embedded.	Obedience	Tradition	UN Tickets	Nepotism	Blood Don.	Trust
					Panel A. Distance					
Distance	0.17***	0.22**	0.32**	-0.40***	-0.85	-0.48**	-0.08	-0.43***	3.50***	0.37***
(in 1,000 km)	(0.04)	(0.08)	(0.12)	(0.07)	(1.31)	(0.15)	(0.11)	(0.09)	(0.79)	(0.11)
N	151	93	71	71	96	71	141	108	138	70
R^2	0.510	0.643	0.417	0.726	0.624	0.275	0.263	0.455	0.643	0.416
					Panel B. Timing					
Timing	-0.07*	-0.05	-0.16*	0.10*	1.23	0.17*	0.14†	0.17**	-0.13	-0.17*
(in 1,000 years)	(0.03)	(0.04)	(0.07)	(0.04)	(0.83)	(0.09)	(0.08)	(0.05)	(0.72)	(0.06)
N	151	93	71	71	96	71	141	108	138	70
R^2	0.475	0.617	0.395	0.641	0.632	0.168	0.277	0.366	0.606	0.369
					Panel C. Distance and Timing					
Distance	0.16***	0.22*	0.25†	-0.42***	0.15	-0.45**	-0.01	-0.39***	3.73***	0.31**
(in 1,000 km)	(0.04)	(0.09)	(0.14)	(0.08)	(1.32)	(0.16)	(0.11)	(0.10)	(0.81)	(0.11)
Timing	-0.03	-0.01	-0.08	-0.02	1.27	0.03	0.14	0.06	0.56	-0.07
(in 1,000 years)	(0.03)	(0.05)	(0.08)	(0.05)	(0.86)	(0.09)	(0.08)	(0.06)	(0.73)	(0.07)
N	151	93	71	71	96	71	141	108	138	70
R^2	0.514	0.643	0.427	0.727	0.632	0.277	0.277	0.461	0.645	0.425

This table reports OLS estimates from regressing country-level psychological outcomes on ancestrey-adjusted Distance to innovation (in 1,000 km) and ancestry-adjusted timing of transition to agriculture (in 1,000 years). Outcome variables are standardized (z-scores). All regressions include the following set of controls: ruggedness, mean distance to waterways, caloric suitability, absolute latitude, and continent fixed effects. Except for distance, data are from SBBH. Robust standard errors are reported in parentheses. VIF values in column 1 of Panel C are 1.88 for Distance and 2.85 for Timing.
†≤ 0.1, * P ≤ 0.05, ** P ≤ 0.01, *** P ≤ 0.001. Significance levels are based on two-tailed tests.

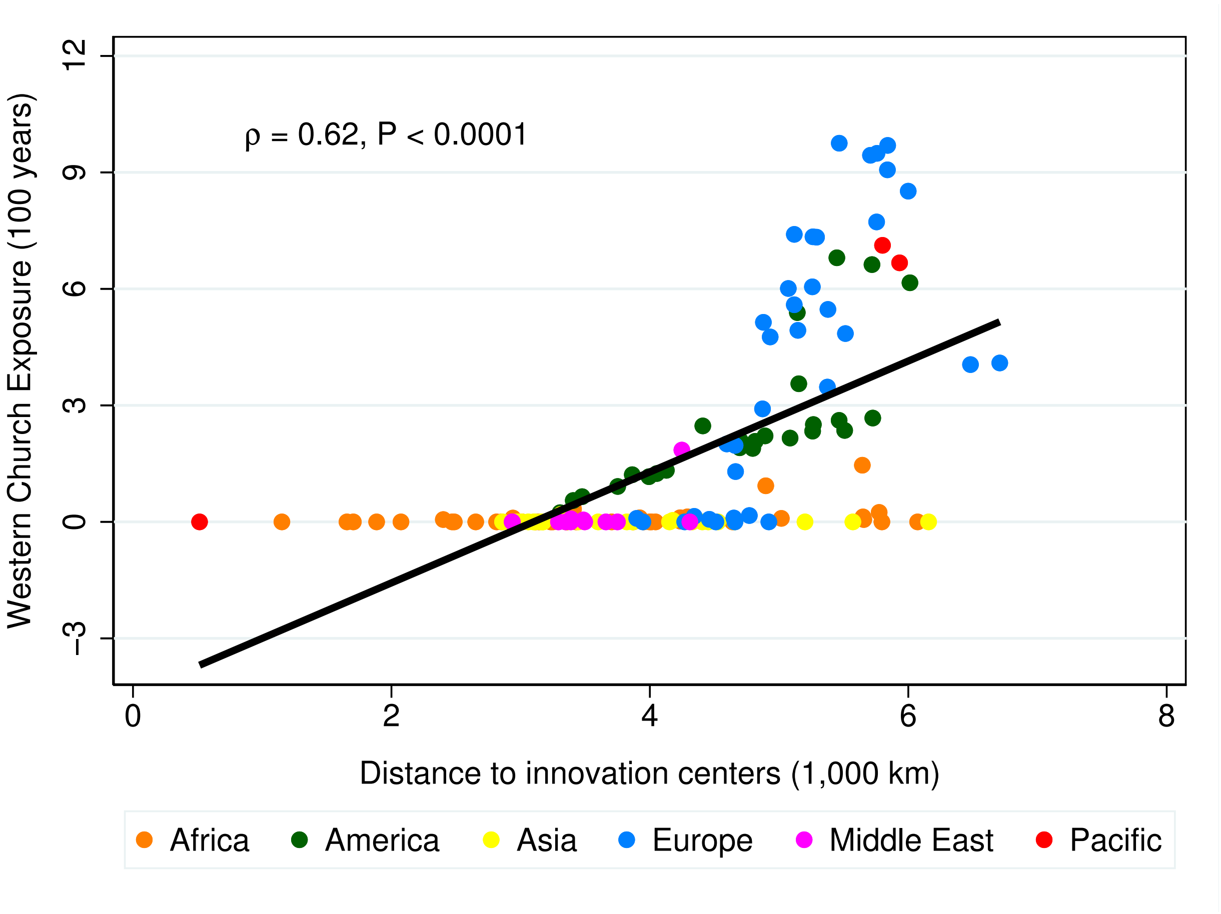

Figure S1. Distance to Innovation and Western Church Exposure

References

Amorim, Carlos Eduardo G., Stefania Vai, Cosimo Posth, and et al. 2018. "Understanding 6th-Century Barbarian Social Organization and Migration Through Paleogenomics." *Nature Communications*, 9: 3547.

Bentley, Alexander R. 2003. "Mobility and the Diversity of Early Neolithic Lives: Isotopic Evidence from Skeletons." *Journal of Anthropological Archaeology*, 32(3): 303–12.

Coleman, Emily R. 1971. "Medieval Marriage Characteristics: A Neglected Factor in the History of Medieval Serfdom." *Journal of Interdisciplinary History*, 2(2): 205–19.

Crook, John A. 1967. *Law and Life of Rome.* Cornell University Press.

Dixon, Suzanne. 1988. *The Roman Mother.* Croom Helm.

Duranton, Gilles, Andrés Rodríguez-Pose, and Richard Sandall. 2009. "Family Types and the Persistence of Regional Disparities in Europe." *Economic Geography*, 85(1): 23–47.

Edmondson, Jonathan. 2005. "Family Relations in Roman Lusitania: Social Change in a Roman Province?" In *The Roman Family in the Empire: Rome, Italy, and Beyond*, edited by Michele George, 183–219. Oxford University Press.

Falkenstein, Frank. 2005. "Aspekte von Alter und Geschlecht im Bestattungsbrauchtum der nordalpinen Bronzezeit." *Alter und Geschlecht in urund frühgeschichtlichen Gesellschaften*, 73–90. Univforsch. Prahist. Arch.

Fisher, D. J. V. 1973. *The Anglo-Saxon Age c.400–1042.* Routledge.

Flannery, Kent V. 2002. "The Origins of the Village Revisited: From Nuclear to Extended Households." *American Antiquity*, 67(3): 417–433.

George, Michele. 2005. "Family Imagery and Family Values in Roman Italy." In *The Roman Family in the Empire: Rome, Italy, and Beyond*, edited by Michele George, 37–68. Oxford University Press.

Ghurye, Govind Sadashiv. 1955. *Family and Kin in Indo-European Culture.* Oxford University Press.

Goetz, Hans-Werner. 2005. "Coutume d'Héritage et Structures Familiales au Haut Moyen Âge." In *Sauver son Âme et se Perpétuer : Transmission du Patrimoine et Mémoire au Haut Moyen-Âge*, edited by François Bourgard, Cristina La Rocca and Régine Le Jan, 203–37. Publications de l'École française de Rome.

Gomes, Cláudia, Gerard Remolins, Ana María López-Parra, Juan F. Gibaja, Maria Fondevila, Flavio De Angelis, Virginia Veltre, M. Eulàlia Subirà, Carlos Baeza-Richer, Diana Guerrero, Sara Palomo-Díez, Cristina Martínez-Labarga, Elena Labajo-González, Maria Victoria Lareu, Bernardo Perea-Pérez, and Eduardo Arroyo-Pardo. 2020. "Paleogenetic Evidence of a Pyrenean Neolithic Family: Kinship, Physical Appearance and Biogeography Multidisciplinary Analysis." *Journal of Archaeological Science*, 123: 105226.

Guichard, Pierre. 1986. "Parenté Indifférenciée et Législation Romano-Barbare." In *La Famille Occidentale au Moyen Âge*, edited by Henri Bresc, Jean-Pierre Cuvillier, Robert Fossier, Pierre Guichard and Pierre Toubert, 83–95. Armand Colin.

Guilaine, Jean. 2016. "Maisons Néolithiques : Exemples Méditerranéens." *Palethnologie*, 8: 468.

Haak, Wolfgang, Guido Brandt, Hylke N. de Jong, Christian Meyer, Robert Ganslmeier, Volker Heyd, Chris Hawkesworth, Alistair W. G. Pike, Harald Meller, and Kurt W. Alt. 2008. "Ancient DNA, Strontium Isotopes, and Osteological Analyses Shed Light on Social and Kinship Organization of the Later Stone Age." *Proceedings of the National Academy of Sciences*, 105(47): 18226–31.

Headland, Thomas N. 1987. "Kinship and Social Behavior Among Agta Negrito Hunter-Gatherer." *Ethnology*, 26(4): 261.

Huebner, Sabine R. 2010. "Household Composition in the Ancient Mediterranean – What do we Really Know?" *A Companion to Families in the Greek and Roman Worlds*, Chapter 4, 73–91. John Wiley & Sons, Ltd.

Johnson, Allen W., and Timothy Earle. 2000. *The Evolution of Human Societies: From Foraging Group to Agrarian State.* Stanford University Press.

King, P. D. 1972. *Law and Society in the Visigothic Kingdom.* Cambridge University Press.

Kuchenbuch, Ludolf. 1978. *Bäuerlich Gesellschaft und Klosterherrschaft im 9. Jahrhundert.* Beiheft.

Lancaster, Lorraine. 1958. "Kinship in Anglo-Saxon Society—I." *The British Journal of Sociology*, 9(3): 230–50.

Lassen, Eva Marie. 1997. "The Roman Family: Ideal and Metaphor." In *Constructing Early Christian Families*, edited by Halvor Moxnes, 103–20. Routledge.

Lee, Richard, and Irven Devore, ed. 1968. *Man the Hunter.* Aldine Transaction.

Le Jan, Régine. 1995. *Famille et Pouvoir dans le Monde Franc (VIIe–Xe Siècle). Essai d'Anthropologie Sociale.* Publications de la Sorbonne.

Martin, Dale B. 1996. "The Construction of the Ancient Family: Methodological Considerations." *The Journal of Roman Studies*, 86: 40–60.

Murray, Alexander Callander. 1983. *Germanic Kinship Structure.* Pontifical Institute of Mediaeval Studies.

Parkin, Tim G., and Arthur John Pomeroy. 2007. *Roman Social History: A Sourcebook.* Routledge.

Peytremann, Edith. 2003. *Archéologie de l'Habitat Rural dans le Nord de la France du IVe au XIIe Siècle.* Association Française d'Archéologie Mérovingienne.

Saller, Richard P. 1994. *Patriarchy, Property and Death in the Roman Family.* Cambridge University Press.

Schultz, Jonathan, Duman Bahrami-Rad, Jonathan P. Beauchamp, and Joseph Henrich. 2019. "The Church, Intensive Kinship, and Global Psychological Variation." *Science*, 366(6466): 1–12.

Shaw, Brent D., and Richard P. Saller. 1984. "Close-Kin Marriage in Roman Society?" *Man*, 19(3): 432–44.

Sjögren, Karl-Göran, Inigo Olalde, Sophie Carver, Morten E Allentoft, Tim Knowles, Guus Kroonen, Alistair WG Pike, Peter Schröter, Keri A Brown, Kate Robson Brown, et al. 2020. "Kinship and Social Organization in Copper Age Europe. A Cross-Disciplinary Analysis of Archaeology, DNA, Isotopes, and Anthropology from Two Bell Beaker Cemeteries." *Plos one*, 15(11): e0241278.

Stuart, David E. 1972. *Band Structure and Ecological Variability : The Ona and Yahgon of Tierra del Fuego.* University of New Mexico.

Theis, Laurent. 1976. "Saints Sans Famille? Quelques Remarques sur la Famille dans le Monde Franc à Travers les Sources Hagiographiques." *Revue Historique*, 255(1): 3–20.

Todd, Emmanuel. 2011. *L'Origine des Systèmes Familiaux. Tome 1 : L'Eurasie.* Gallimard.

Toubert, Pierre. 1976. *Les structures du Latium Médiéval: Le Latium Méridional de la Sabine du IXe Siècle à la fin du XIIe Siècle.* École Française de Rome.

Wallace-Hadrill, Andrew. 1994. *Houses and Society in Pompeii and Herculaneum.* Princeton University Press.

Wickham, Chris. 1982. *Studi Sulla Società Degli Appennini Nell'alto Medioevo.* Univrsità Degli Studi di Bologna.

Zerner, Monique. 1981. "Enfants et Jeunes au IXe Siècle : La Démographie du Polyptyque de Marseille 813–814." *Provence Historique*, 126: 355–84.
